# Genomic characterization of Pseudomonas aeruginosa from canine otitis highlights the need for a One Health approach to this opportunistic pathogen

**DOI:** 10.1099/mgen.0.001407

**Published:** 2025-05-01

**Authors:** L. Newstead, T. Smith-Zaitlik, C. Kelly, E. Roberts, S. Street, G.K. Paterson

**Affiliations:** 1 Royal (Dick) School of Veterinary Studies and the Roslin Institute, University of Edinburgh, Easter Bush, Midlothian, Edinburgh, EH25 9RG, UK

**Keywords:** bacterial drug resistance, bacterial genome, canine, One Health, otitis, *Pseudomonas aeruginosa*

## Abstract

In humans, *Pseudomonas aeruginosa* is well known as a prominent opportunistic pathogen associated with antimicrobial resistance (AMR), which presents a major challenge to successful treatment. This is also the case in animals, particularly in companion dogs where *P. aeruginosa* is a common cause of otitis. Despite its clinical significance, little data are available on the genomics and epidemiology of *P. aeruginosa* in dogs. To address this, we have genome-sequenced 34 canine otitis *P. aeruginosa* isolates from a veterinary referral hospital and analysed these along with a further 62 publicly available genomes from canine isolates. Phylogenetic analysis revealed that all three *P. aeruginosa* phylogroups*,* A–C*,* are represented amongst a diverse bacterial population isolated from dogs. We identify examples of persistent or recurrent infection by the same strain of up to 309 days between sampling, demonstrating the difficulty of successfully eradicating infection. Isolates encoded a variety of AMR genes with genomic and phenotypic AMR correlating poorly for β-lactams but showing complete concordance between fluoroquinolone resistance and quinolone resistance-determining regions (QRDRs) of DNA gyrase and topoisomerase IV. Pangenome-wide analysis between 80 canine otitis isolates (34 newly sequenced here and a further 46 publicly available) and a reference collection of 491 human isolates found no genes which were over-represented or specific to either host species, indicating similar strains infect both humans and dogs. This agrees with the sharing of multilocus sequence types between dogs and humans, including the isolation here of ST235 from three dogs, a lineage prominent among the multidrug resistant (MDR) and extensively drug-resistant (XDR) international high-risk clones of *P. aeruginosa* causing human infections. The presence of such ‘high-risk' clones in companion dogs is concerning given their potential impact on animal health and the potential for zoonotic spread. These data provide new insight into this difficult-to-treat veterinary pathogen and promote the need for a One Health approach to tackling it.

**Impact Statement**

*Pseudomonas aeruginosa* is a major human pathogen associated with antimicrobial resistance. This is also the case in small-animal medicine, particularly as a cause of intractable canine otitis. Little is known about the genomics and epidemiology of strains that cause canine infections, and this study presents an analysis of 34 newly sequenced *P. aeruginosa* isolates from canine otitis. These data are important for future surveillance, particularly for the monitoring of antimicrobial resistance, ‘high-risk’ clones and zoonotic infection. They also provide a basis to better understand disease pathogenesis, which can underpin the development of novel diagnostics and interventions. The recognition here that similar strains are infecting both humans and dogs emphasizes the need for a One Health approach in tackling *P. aeruginosa.*

## Data Summary

The authors confirm that all supporting data, codes and protocols have been provided within the article or through supplementary data files.

## Introduction

*Pseudomonas aeruginosa,* a Gram-stain-negative bacterium, found throughout the environment, is clinically significant as a common opportunistic pathogen in humans [1,4]. In particular, *P. aeruginosa* is an important pathogen among patients with cystic fibrosis, often causing chronic respiratory infections [3]. *P. aeruginosa* infections can be extremely difficult to treat as they are often resistant to conventional antimicrobials due to high intrinsic antimicrobial resistance (AMR) and the ability to develop new resistance in response to antimicrobial treatment [5]. Indeed, *P. aeruginosa* is designated by the World Health Organisation as a ‘priority pathogen’ for the development of new antimicrobial agents [6] and is a member of the ESKAPE pathogens (*Enterococcus faecium*, *Staphylococcus aureus*, *Klebsiella pneumoniae*, *Acinetobacter baumannii*, *Pseudomonas aeruginosa* and *Enterobacter* spp.) comprising the most challenging multidrug-resistant nosocomial pathogens [7]. In 2019, *P*. aeruginosa was estimated to be responsible for 43,801 deaths, of which 10,900 were attributable to AMR [8].

The challenges of *P. aeruginosa* infections and AMR are not limited to human medicine as this bacterium is able to cause a range of infections in other animals [9,11]. In companion dogs, it is a notable opportunistic pathogen, predominantly in otitis [12,14], but it is also associated with other conditions including pyoderma [15], ulcerative keratitis [16] and urinary tract infections [17]. In canine otitis, *P. aeruginosa* can be isolated from 28 to 37% of cases [18,20] and is especially prevalent in chronic and intractable cases [21,22]. In contrast, the bacterium is rarely present in healthy dog ears [21,23,25]. A large study of veterinary practice data in England reported otitis externa as the most frequent disorder in dogs, with a prevalence of 10.2% [26]. Canine otitis externa is known to have a significant negative impact on the welfare of both dogs [27] and owners [28,29], and the condition has been ranked as the second-most severe disorder in dogs by a primary veterinary care study [30].

Alongside *Staphylococcus pseudintermedius* and *Escherichia coli*, *P. aeruginosa* has been identified by the European Food Safety Authority Panel on Animal Health and Welfare as the most relevant AMR bacteria in the European Union [31]. Canine otitis caused by *P. aeruginosa* comprises a notable part of the One Health challenge posed by this opportunistic pathogen, necessitating considerable antimicrobial use and presenting a zoonotic risk, in addition to animal welfare concerns raised. While *P. aeruginosa* from human infections are well studied, there is little literature available on the molecular epidemiology and genomics of *P. aeruginosa* isolates from canine infections. We have, therefore, investigated the population structure, AMR and relationship to human isolates of *P. aeruginosa* from canine otitis from a veterinary referral hospital in the UK.

## Methods

### Isolate collection and whole-genome sequencing

Canine otitis samples from referral patients at the Hospital for Small Animals, Royal (Dick) School of Veterinary Studies (R(D)SVS), University of Edinburgh were cultured on Columbia blood agar with 5% (v/v) horse blood (E and O Laboratories Ltd.) by the Easter Bush Pathology microbiology laboratory as part of their routine diagnostic service. Isolates were identified using Vitek2™ (bioMérieux) following the manufacturer’s guidelines. For this study, all 34 otitis isolates identified as *P. aeruginosa* between 01 July 2017 and 31 October 2022 were genome sequenced. Whole-genome sequencing was performed by Microbes NG (University of Birmingham, UK) as described previously [32]. Contigs<200 bp were removed manually, and contaminant sequences were removed automatically during upload to NCBI via the NCBI Foreign Contamination Screen [33]. CheckM analysis (v1.2.2) was also performed during upload to NCBI to assess genome assembly quality with respect to percentage completeness and contamination [34]. Taxonomy was confirmed during upload to NCBI using average nucleotide identity (ANI) [35] and independently by digital DNA:DNA hybridization via the Type Strain Genome Server [36]. Strain metadata and accession are presented in Table S1 (available in the online version of this article).

### Phylogenetic and pangenome analyses

#### Quality control and inclusion of additional *P. aeruginosa* genomes

Sixty-two additional canine *P. aeruginosa* genomes were downloaded from the NCBI assembly database (https://www.ncbi.nlm.nih.gov/assembly; National Centre for Biotechnology Information, Maryland, USA) (Table S2). Genomes were included if the biosample entry or an associated paper contained the following associated data: host species, country of isolation and sample type or host disease.

The quality of the *P. aeruginosa* genome assemblies was assessed using QUAST v5.2.0 [37], and the output was compiled with multiQC v1.13 [38]. Draft genome assemblies were annotated using Prokka v1.14.6 with default parameters [39]. As a further measure of quality control, the whole-genome average nucleotide identity was calculated for all-v-all genome assemblies with fastANI v1.33 [40] to identify any genome assemblies that may contain contaminant sequences based on a threshold of ≥95 % ANI for delineating species [41].

#### Alignment and phylogenetic analysis

The Snippy v4.6 pipeline (https://github.com/tseemann/snippy) was used to perform variant calling and produce core genome alignments of the *P. aeruginosa* isolates against reference genomes PA01 (accession GCF_000006765.1_ASM676v1) and PA14 (accession GCF_000014625.1_ASM1462v1). Additionally, a pangenome analysis was carried out for the canine *P. aeruginosa* genomes and reference genomes PAO1 and PA14 with PIRATE (Pangenome Iterative Refinement and Threshold Evaluation) v1.0.5 [42], using default parameters as follows: CoDing Sequence (CDS) used as feature pangenome construction, paralog classification enabled and identity thresholds used for pangenome construction 50, 60, 70, 80, 90, 95 and 98%. The core genome was defined as CDS present in ≥95% of genomes, and accessory CDS as those present in <95 % but at least one genome. Using the core genome alignments produced against reference genomes PAO1 and PA14 and the core gene alignment produced with PIRATE, maximum-likelihood (ML) phylogenetic trees were inferred with IQtree v2.2.2.3 [43], using the general time reversible (GTR) substitution model and 1,000 UltraFast bootstraps [44]. Phylogenetic trees were visualized, annotated and rooted with iTOL v5 [45].

#### Pangenome-wide-association analysis

Further pangenome analysis was carried out with PIRATE v1.0.5 [42], using default parameters as outlined above, to include *P. aeruginosa* isolates from human infections, alongside 80 canine isolates obtained from otitis cases (34 newly sequenced and 46 from NCBI). The genomes of 491 human *P. aeruginosa* isolates were downloaded and included in the subsequent pangenomic analysis (all canine and human isolates in the analysis are presented in Table S3). These isolates comprised PA01, all 103 UK human isolates available from the International Pseudomonas Consortium Database ((https://ipcd.ibis.ulaval.ca/index) when accessed on 15 November 2023) [46] and 387 isolates from an international dataset (BioProject PRJNA264310) [47]. That latter dataset consisted of 390 isolates in the original publication [47], but NCBI filtered out three entries that did not have the requested ReleaseType or were suppressed. A pangenome-wide gene association analysis was carried out with Scoary v1.6.16 [48], to identify genes over-represented or specific to either of the host species (human or canine), using the gene presence–absence matrix output from PIRATE as the input for Scoary.

### Genome analysis for MLST, AMR genes and bacteriocin genes

Multilocus sequence typing (MLST) was performed on genome sequences using PathogenWatch (https://pathogen.watch/) [49], and two novel sequence types (STs) were allocated via genome submission to https://pubmlst.org/ [50]. Abricate v1.0.1 (https://github.com/tseemann/abricate) was used to screen for AMR genes utilizing the CARD database [51,52] (database versions from 27 March 2021) with genes being identified using thresholds of ≥80 % nucleotide sequence identity and ≥80 % query sequence coverage. AMR gene presence and absence was aligned to the study isolate bacterial phylogeny using ggTree v3.10.0 [53] in RStudio v4.3.2 [54]. Bacteriocin genes encoded in the 34 UK study isolates were identified using BAGEL4 [55].

### Phenotypic antimicrobial susceptibility testing

Antimicrobial susceptibility testing was performed using disc diffusion following the 2023 European Committee on Antimicrobial Susceptibility Testing guidelines [56] and breakpoints [57]. *P. aeruginosa* ATCC 27853 was used for routine quality control, and *Escherichia coli* ATCC 35218 and *Klebsiella pneumoniae* ATCC 700603 were included for control of the inhibitor component of β-lactam–inhibitor combinations [58]. All control isolates produced zones of inhibition within the acceptable ranges [58]. The antimicrobials tested are presented in Table S4. AMR phenotypic and genomic concordance/discordance was determined by comparing genomic data (i.e. presence or absence of AMR genes/mutations) with phenotypic disc diffusion testing data for the 34 R(D)SVS *P. aeruginosa* isolates against 10 antimicrobials (*n*=340 combinations) as described previously [59], with full results presented in Table S5.

## Results and discussion

### MLST and phylogenetic analysis of canine otitis *P*. *aeruginosa*

Thirty-four otitis isolates of *P. aeruginosa* cultured from canine ear swabs and fluid at the R(D)SVS were genome sequenced using Illumina technology. Their genome varied in length from 6.26 Mp to 7.00 Mp with a mean of 6.58 Mp (Table S1), consistent with previously reported genome lengths of *P. aeruginosa* isolated from clinical and environmental settings [60].

Twenty-five STs were represented among the 34 canine isolates (Table S1). These came from 30 individual dogs, with four dogs providing two isolates, three dogs providing one isolate from two sampling time points, and a fourth dog with isolates being collected at the same time but from different ears. In each of these four cases, the same ST was found in both isolates; thus, 25 STs were isolates from 30 individual dogs. The most frequent of those being ST27 and ST235 (three isolates each), another five STs were found in two isolates, while the remaining 19 STs were found in single isolates. Two new STs were found: ST4532 in isolate 234181_R, which comprised a novel combination of previously recognized alleles and ST1244, a previously assigned ST, with no corresponding isolate representing it. Previous work has isolated a range of *P. aeruginosa* STs from dogs, and our study provides agreement that diverse isolates are responsible for canine infections [10,61]. The isolation of ST235 from three individual dogs is noteworthy, given that this lineage is among the MDR/XDR international high-risk clones of *P. aeruginosa* causing human infections [62,63]. Albeit two of the three study isolates were pan-susceptible to the antimicrobials tested here, with the third one resistant only to certain β-lactams. It is the only example of a high-risk clone among the canine study isolates here, although the high-risk clones, ST111, ST233 and ST244, have also been isolated from dogs [64,65]. ST235 has a wide-ranging international distribution in human disease and is not only associated with AMR but also with increased virulence and mortality in human patients [66,69]. The ExoU cytotoxin encoded by ST235 likely contributes to this increased virulence [70,71], and all three canine study isolates were *exoU*-positive. The presence of ST235 and other high-risk clones in companion animals should be closely monitored given the risk that they may pose to the animals and owners alike. An analysis of the *P. aeruginosa* PubMLST database (https://pubmlst.org/organisms/pseudomonas-aeruginosa accessed 24.03.25) [50] reveals that 20 out of the 25 STs identified among study isolates have been previously found in human disease, supporting the suggestion that similar *P. aeruginosa* strains can cause infections in both humans and dogs. Of the remaining five STs, two are new from this study and have not yet been reported from elsewhere. The three others, ST2459, ST2460 and ST3690, have only been isolated from dogs, but in each case, only a single isolate is documented on the PubMLST database. Therefore, any association between these STs and dogs will require the isolation of further isolates.

**Fig. 1.**
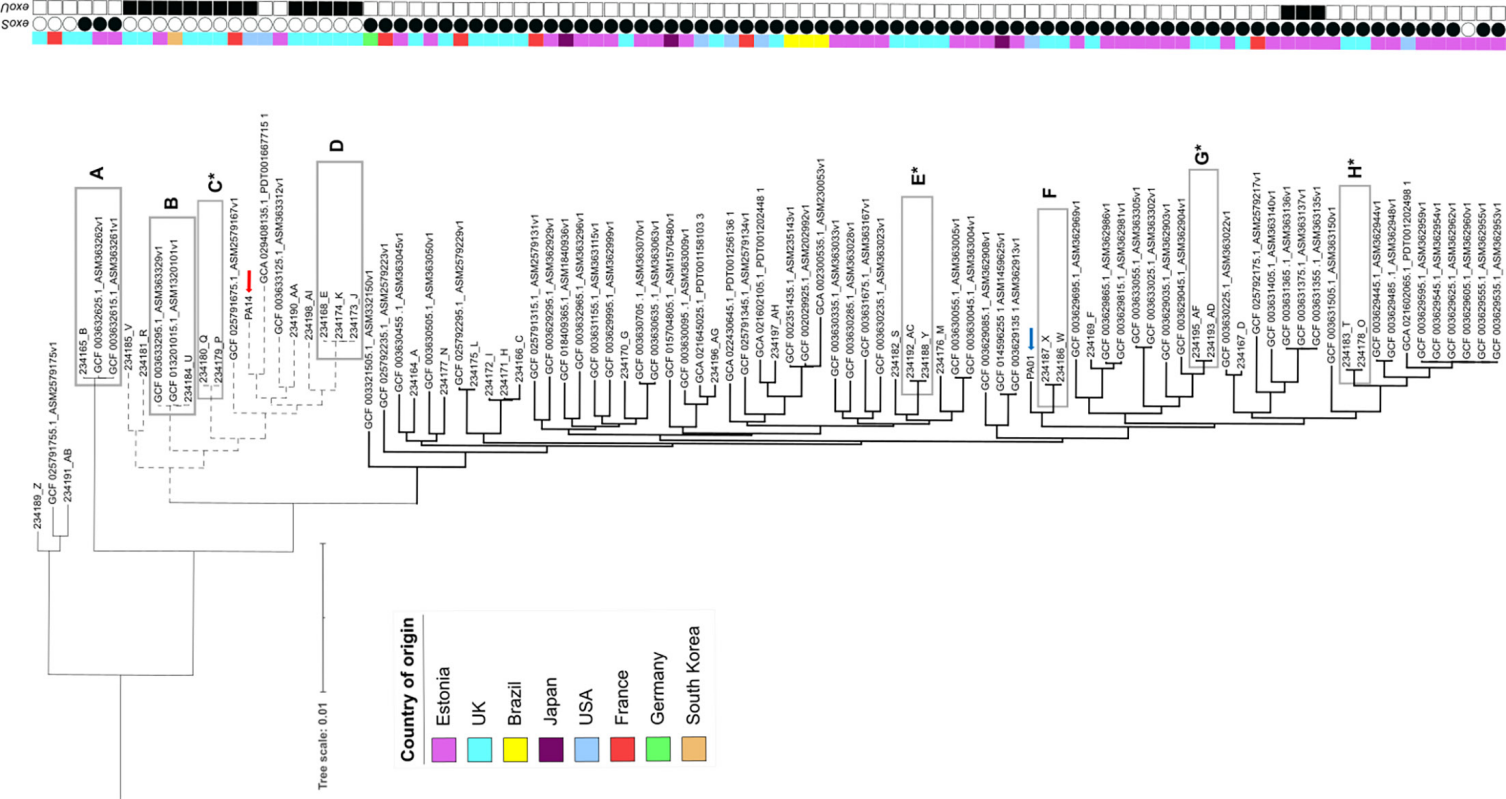
Mid-point rooted phylogeny of canine *P. aeruginosa* isolates constructed from core genome SNPs derived from the pangenome of all isolates included in the phylogeny; coloured bar indicates country of origin. Boxed isolates, A–H, indicate closely related isolates, where at least one of those isolates is from this study. Clades most closely related to either PA01 (phylogroup 1) or PA14 (phylogroup 2) reference strains are indicated by bold and dashed branches, respectively. For orientation, PA01 and PA14 are indicated by blue and red arrows, respectively. The presence and absence of *exoU* and *exoS* are indicated by filled and empty symbols, respectively. Most isolates from the R(D)SVS came from individual dogs, the exceptions being four separate animals providing two isolates each. In these cases, the pair of isolates was closely related, and the boxes C, E, G and H are indicated by an asterisk. Further isolate details are provided in Tables S1 and S2.

A mid-point rooted phylogeny of canine *P. aeruginosa* isolates was constructed from core genome SNPs derived from the pangenome of all isolates included in the phylogeny (Fig. 1). This approach was used to avoid any potential bias caused by the use of a single reference strain for SNP analysis. Although phylogenies were also produced using either PA01 or PA14, which produced similar results (data not shown). A further 62 canine *P. aeruginosa* genomes available from the NCBI assembly database were included for comparison with R(D)SVS isolates (Table S2). These came from Estonia (42 isolates), France (7 isolates), USA (5 isolates), Brazil (3 isolates), Japan (3 isolates) and Germany and South Korea (one isolate each). The majority of these additional canine isolates came from auricular sites (77%, 44/62), and the other isolates came from a variety of sites, most predominately from skin/wound (eight isolates). This phylogeny indicated a diverse population of canine isolates with a mean pairwise SNP distance of 49,536 between UK study isolates and 41,294 between all 96 canine *P. aeruginosa* isolates (with PA01 and PA14 reference strains removed from pairwise calculations) (Table S6). The lower average value for the whole collection reflects the inclusion of several highly related isolates among those sequenced from Estonia.

The *P. aeruginosa* population has previously been classified into three phylogroups: A containing reference strain PAO1, B containing PA14 and a third minor group, C [47,72, 73]. Phylogroups A and B are further distinguished by the distribution of *exoU* and *exoS*, which encode effector proteins secreted by the *P. aeruginosa* type III secretion system. *exoS* is highly overrepresented in phylogroup A compared with phylogroup B, and *exoU*, conversely, is highly overrepresented in phylogroup B compared with phylogroup A [73]. The majority of canine isolates (75/96) were more closely related to PA01 than to PA14, indicating that most isolates belong to the *P. aeruginosa* phylogenetic group A. Fifteen other canine isolates were more closely related to PA14 and, therefore, likely represented phylogenetic group B. Consistent with this assignment to phylogenetic groups was the distribution of *exoU* and *exoS* (Fig. 1). *exoU,* indicative of phylogenetic group B, was present in 13 out of 15 canine isolates that are more closely related to PA14 and only present in 3 of the other 81 isolates. Conversely, *exoS* was absent in all 15 of those PA14-related isolates but was present in 74 of 75 PA01-related isolates. A further six canine isolates, including three from R(D)SVS, did not appear to be closely related to either reference strain and likely represent phylogenetic group C isolates.

There were several examples of closely related isolates involving R(D)SVS study isolates (Fig. 1 Boxes A-H). In four of these cases, the two related isolates came from the same individual dog (Fig. 1 Boxes C, E, G and H). Isolates 234179_P and 234180_Q came from different ears from the same dog on the same day and are separated by 47 SNPs (Fig. 1 Box C). Isolates 234193_AD and 234195_AF came from another individual dog 96 days apart and are separated by 193 SNPs (Fig. 1 Box G). Isolates 234178_O and 234183_T are also from another individual animal and separated by 53 SNPs but were isolated 169 days apart from the same ear (Fig. 1 Box H). 234188_Y and 234192_AC (Fig. 1 Box E) were isolated from another individual 309 days apart and are separated by 11 SNPs. These last three examples illustrate the difficulty in successfully clearing *P. aeruginosa* otitis infections and confirm the persistence of the same strain during chronic or recurrent infection.

Closely related UK isolates were not restricted to the examples of isolates collected from the same individual dogs. Among the 34 UK isolates, there are two examples of closely related isolates (Boxes D and F, Fig. 1) cultured from different dogs. Isolates 234168_E, 234173_J and 234174_K (Box D, Fig. 1) came from three different dogs, with the latter two isolates separated by 60 SNPs, whereas isolate 234168_E was more distinct with an SNP distance of 417–477 from those other two. These are notable as being the three isolates belonging to ST235. Isolates 234186_W and 234187_X (Box F, Fig. 1), also from different dogs, were collected 75 days apart and were separated by ten SNPs. While epidemiological links between these animals have not been established, the closely related strains may indicate potential common sources or transmission events that, if detected in near real time, would merit further surveillance and investigation.

There are also two examples of UK study strains closely related to isolates from other countries. 234165_B was separated by 413 and 419 SNPs from two Estonian isolates (Fig. 1 Box A), while isolate 234184_U was 262 and 486 SNPs from Estonian and South Korean isolates, respectively (Fig. 1 Box B). While there is insufficient metadata to consider any potential epidemiological links of these related isolates, it does suggest that there are instances of international dissemination of clones causing canine otitis.

### Phenotypic and genotypic antimicrobial susceptibility

Nineteen out of 34 isolates (55.9%) were susceptible to all ten tested antimicrobials with 15 (44.1%) showing resistance to at least one (Table S4). Resistance was most frequent against the quinolones: ciprofloxacin and levofloxacin. Twelve isolates (35.3%) were resistant to both of these antimicrobials, with all other isolates being susceptible to both. No isolates were classed as multidrug resistant, i.e. acquired non-susceptibility to at least one agent in three or more antimicrobial categories. Although seven isolates showed acquired resistance to two antimicrobial categories. Five isolates (14.7%) were resistant to aztreonam and three (8.8%) were resistant to cefepime. Two isolates (5.9%) were resistant to both aminoglycosides tested, amikacin and tobramycin, and a single isolate (2.9%) was resistant to piperacillin/tazobactam. No resistance was seen for the two carbapenems, meropenem and doripenem, nor the cephalosporin ceftazidime.

**Fig. 2.**
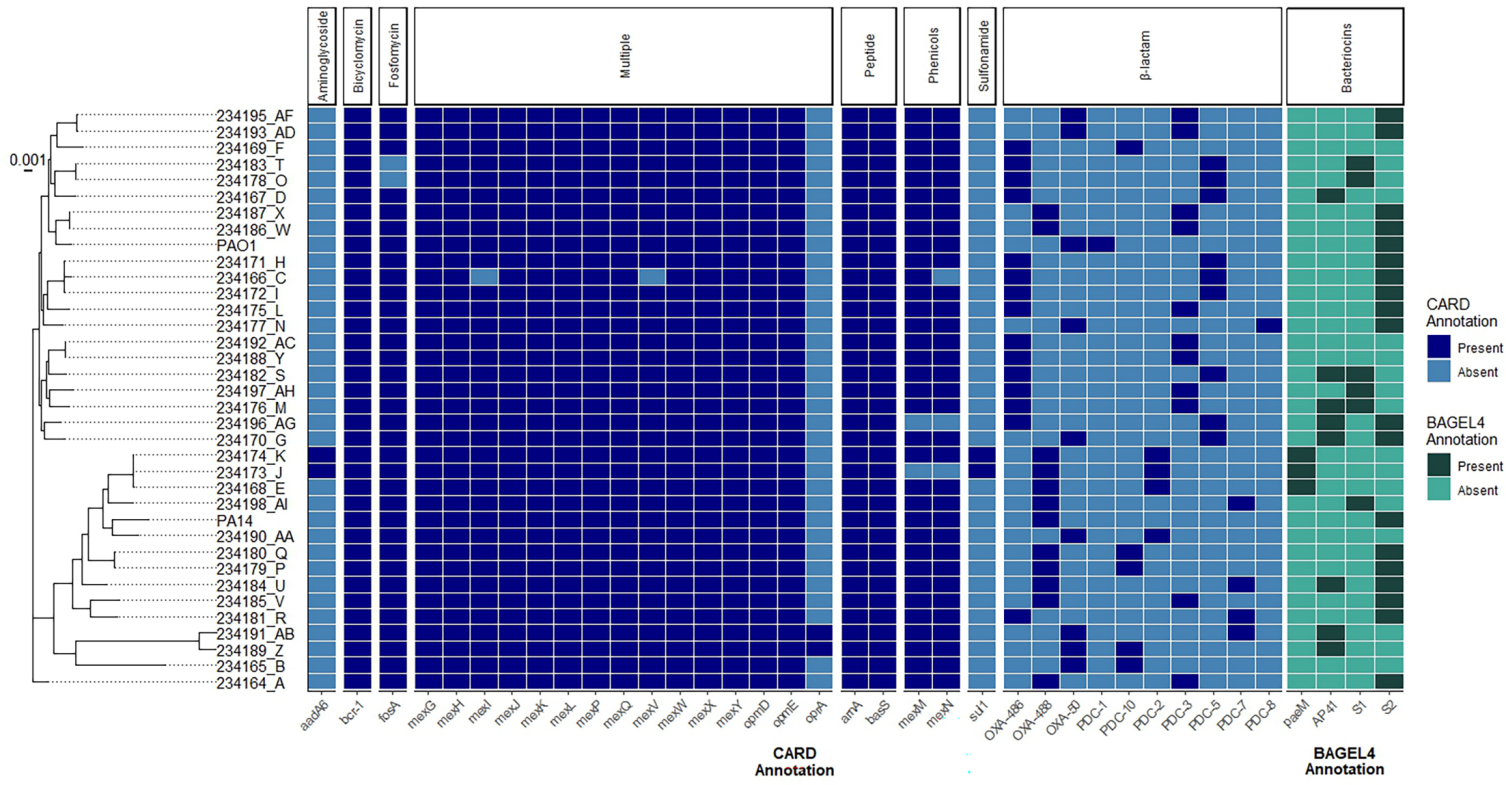
Predicted antimicrobial resistance and bacteriocin genes among 34 study *P*. aeruginosa otitis externa isolates and PA01 and PA14 (reference strains). Pairs of isolates from the same individual animals possessed the same gene profile. Gene presence (dark blue) and absence (light blue) was visualized using ggTree v3.10.0 in RStudio v4.3.2.

Using RGI/CARD (Resistance Gene Identifier/Comprehensive Antibiotic Resistance Database), a total of 57 unique genes associated with AMR were identified among the 34 study isolates (Fig. 2). The number of such genes in individual isolates ranged from 41 to 47. A majority of these genes (36/57, 63.2%) were present in all isolates regardless of differences in phenotypic resistance. For the tested β-lactams, there was a poor concordance (3.92%) between resistance genotype and phenotype (Table S5). Concordance between genotype and phenotype improved to 88.24 % for aminoglycoside sensitivity testing, but most isolates (30/34) were sensitive and lacked resistance genes, while in a few instances of phenotypic (*n*=2) or genotypic (*n*=2) resistance, there was complete discordance. Thus, further testing with a wider range of phenotypes and genotypes, and combinations thereof, would be required to confidently ascertain the concordance between genotype and phenotype in the case of aminoglycosides. Discordance between AMR genes and AMR phenotype in *P. aeruginosa* has been reported in previous studies on human clinical isolates [59,74, 75] and is apparent in canine isolates also, particularly for β-lactams. The difficulty in predicting AMR phenotype from genome sequences in *P. aeruginosa* has been ascribed to mutations altering expression levels of efflux pumps, outer membrane proteins and intrinsic β-lactamases [76]. Some disagreement may also arise from inaccuracies in phenotypic testing. In contrast to acquired AMR genes, there was a complete concordance between resistance to quinolones (ciprofloxacin and levofloxacin tested here) and mutations in the quinolone resistance-determining region (QRDR) of DNA gyrase and topoisomerase IV. Most strikingly, all 12 resistance isolates had the Thr-83-Ile mutation in DNA gyrase subunit A, encoded by *gyrA,* while all sensitive isolates had the wild-type residue at this position. The resistant isolates 234171_H and 234193_AD also had a Ser-87-Leu mutation in the A subunit of topoisomerase IV, encoded by *parC,* which was absent in all other isolates. These are two of the most frequently observed mutations linked to quinolone resistance in human clinical *P. aeruginosa* isolates and those exposed to quinolones in the laboratory and have been demonstrated to decrease the binding affinity of quinolones to these targets of action [77,78]. The presence of QRDR mutations in both DNA gyrase subunit A and topoisomerase IV increases quinolone resistance compared to mutation of either protein singly [79]. Only one mutation in the QRDR of DNA gyrase subunit B (amino acid positions 429 to 585), encoded by *gyrB*, was present, an E468D mutation in the resistant isolate 234171_H. Two mutations were noted in the QRDR of topoisomerase IV subunit B (amino acid positions 357 to 503), encoded by *parE*. A I436L in the resistant isolate 234197_AH and V460G in susceptible isolate 234177_N.

### Bacteriocin gene content

*P. aeruginosa* is known to encode a range of bacteriocins (pyocins), small ribosomally encoded peptides with antibacterial activity against other strains or species which are often closely related to the producer [80]. Bacteriocins are purported to provide an ecological advantage against competitors in addition to being of interest as novel antibacterials [81,82]. How they influence *P. aeruginosa* disease pathogenesis in the canine host remains unclear, but they could antagonize the normal microflora in the ear and skin and facilitate colonization and subsequent infection. The majority (29/34, 85.3%) of UK study isolates encoded at least one bacteriocin gene, with two of those predicted to have two such genes (Fig. 2). Five isolates were predicted not to encode a bacteriocin. Four different bacteriocins were detected, all belonging to the S-type pyocins, S2, S1, AP41 and PaeM, with pyocin S2 the most frequent, being found in half of the 34 isolates (Fig. 2).

### Pangenome-wide-association analysis

Host specificity or adaptation of canine and human isolates was examined by pangenome-wide-association analysis to identify genes associated with either host species. This analysis, comprising 80 canine (specifically those from otitis) and 491 human isolates, did not identify any such genes, indicating a lack of host adaptation or specificity in the strains infecting humans and dogs (Tables S37 and 8). This agrees with the same STs being present among human and canine isolates and the recent finding of there being no specific virulence profile associated with canine otitis infection when comparing isolates from human and environmental sources by hierarchical clustering [64]. Further supporting a lack of host specificity among human and canine isolates is the case report of zooanthroponotic transmission between owner and dog of a carbapenemase-producing *P. aeruginosa* [65].

## Conclusion

Infections caused by *P. aeruginosa* are a major challenge in human and veterinary medicine. To address the scarcity of genomic-level insight into the epidemiology of canine *P. aeruginosa,* we have analysed 34 newly sequenced isolates from otitis. This information is valuable in better understanding the pathogenesis of these infections, the design of diagnostics and therapeutics and for the surveillance of a key AMR pathogen. The finding that similar strains infect both human and canine hosts emphasizes the need for a One Health approach when considering *P. aeruginosa* epidemiology and interventions.

## Supplementary material

10.1099/mgen.0.001407 Uncited Supplementary Material 1.

## Abbreviations

AMR: antimicrobial resistance
CDS: CoDing sequence
ESKAPE: *Enterococcus faecium*, *Staphylococcus aureus*, *Klebsiella pneumoniae*, *Acinetobacter baumannii*, *Pseudomonas aeruginosa* and *Enterobacter* spp.
MDR: multidrug resistant
PIRATE: pangenome iterative refinement and threshold evaluation
QRDR: quinolone resistance-determining region
R(D)SVS: Royal (Dick) School of Veterinary Studies
RGI/CARD: resistance gene identifier/comprehensive antibiotic resistance database
XDR: extensively drug-resistant
